# Associations of migraines with suicide ideation or attempts: A meta-analysis

**DOI:** 10.3389/fpubh.2023.1140682

**Published:** 2023-03-24

**Authors:** Huijie Wei, Yu Li, Hua Lei, Junwei Ren

**Affiliations:** ^1^Department of Pathology, Chongqing University FuLing Hospital, Chongqing, China; ^2^Department of Neurology, Chongqing University FuLing Hospital, Chongqing, China

**Keywords:** migraine, suicide ideation, suicide attempt, meta-analysis, association

## Abstract

**Objective:**

Whether migraine is associated with a higher risk of suicide ideation and/or attempts remains controversial. Therefore, we aimed to evaluate these potential associations in migraine patients by performing a meta-analysis of previously published data.

**Methods:**

We searched for studies published up to 31 June 2022 that compared the risk of suicide ideation/attempt in migraineurs and non-migraineurs in PubMed, EMBASE, and Web of Science databases. Sixteen studies fulfilled the eligibility criteria. We applied Random-effects models to calculate pooled adjusted odds ratios (AORs) and 95% confidence intervals (CIs) in patients with migraine.

**Results:**

Migraine patients were at a significantly increased risk of suicide ideation (AOR 1.33, 95% CI 1.15–1.54) and suicide attempts (AOR 1.70, 95% CI 1.42–2.03). The increase in risk may be greater in adults (>19 years) than in younger individuals.

**Conclusion:**

The available evidence indicates a significant association of migraines with suicide ideation and attempts. Future work should confirm and extend these findings, as well as explore whether they are affected by ethnicity or geography.

## Background

Migraines, the second most common primary headache, are recurrent disabling headaches associated with neurologic symptoms, including severe throbbing head pain, with or without visual disturbances (1). According to the Global Burden of Disease Study, migraine is the third leading cause of disability in people under 50 years, and it is the most common neurological disorder, with an estimated global prevalence ranging from 11 to 23% (2, 3). Sufferers of migraine often have a lower health-related quality of life than non-migraineurs, as well as influence work productivity and in social and family relationships (4–6). Migraines are known to be comorbid with other neurological and psychiatric disorders. Of these, about one-third of migraineurs have fibromyalgia (7). Migraine also overlaps with epilepsy in terms of visual and sensory disturbances, pain and altered consciousness (8). And，young women with a history of migraine with aura are associated with acute ischemic stroke (9). Further, migraines increase the risk of depression and post-traumatic stress disorder (10). Therefore, anticipatory identification of possible co-morbidities accompanying migraine is important, which contributes to primary and secondary prevention of co-morbidities and reduces the associated disease burden.

Suicide ideation (thoughts of engaging in behavior intended to end one’s life) and suicide attempts (potentially self-injurious behavior in which there is at least some intent to die) are strongly predictive of suicide deaths which accounted for 1.4% of all deaths (11, 12). Globally, lifetime prevalence rates are ~9.2% for suicide ideation and 2.7% for suicide attempts in the general population, which can lead to injury, hospitalization, and loss of freedom, and which place a financial burden of billions of dollars on societies (13–15). Some studies support that migraineurs are at increased risk of suicide ideation (16–21) and suicide attempts (19, 22–24). However, other work showed that migraine was not associated with suicide ideation (25–27) or suicide attempts (13, 25, 28, 29). The latest meta-analysis indicated a high prevalence of suicide ideation and attempts in migraine patients but the association between them was not specified (30). A previous meta-analysis found that patients with migraine had a higher risk of suicide ideation (31), but more recent studies emerged (17, 25, 26) and reached an opposite conclusion (25, 26).

Given these divergent results, we conducted a literature review and meta-analysis to gain a more complete understanding of the potential link of migraines with suicide ideation and attempts.

## Methods

This meta-analysis was conducted in accordance with the Preferred Reporting Items for Systematic Reviews and Meta-analyses (PRISMA) statement (32) (Supplementary Table 1). The meta-analysis has been registered on the INPLASY website under the registration number INPLASY202330019 (Supplementary file 1).

### Study search and selection strategy

We searched PubMed, EMBASE, and Web of Science databases using the keywords “migraine or headache” and “suicide or suicides” and included articles published until 31 June 2022 (Supplementary file 2). Search results were screened for potentially relevant studies by title and abstract, followed by full-text review and selection. Studies were included if they fulfilled the following requirements: (a) they were observational studies, including cohort, case–control, or cross-sectional studies, evaluating associations of migraines with suicide ideation or attempts; (b) they reported adjusted odds ratios (AORs) with 95% confidence intervals (CIs) in the migraine cohort, and compared the results with a non-migraineur population, and (c) they were published in English. If population in two studies overlapped, only the large study was included. Exclusion criteria during the abstract review included: (a) articles that were meeting abstracts, case reports, discussions, editorials, reviews, letters, or commentaries; or (b) articles analyzing migraine in pregnant women.

### Data extraction

Data were independently extracted from the selected studies by two investigators (Wei HJ and Li Y). If a discrepancy was encountered, a third author (Ren JW) reviewed the article and an agreement was reached. The following data were extracted from all articles: authors, year of publication, country of origin, study design, total sample size, criteria used to assess suicide ideation or attempts, criteria for migraine diagnosis, stratification by migraine subtypes (with or without aura), and potential confounders in the adjusted analysis.

### Statistical analysis

We used Stata version 12.0 (StataCorp, College Station, TX, United States) statistical software to generate summary statistics and pooled AORs using a random model. In studies that evaluated AORs for migraine stratified by subtypes, the lower AORs were used for the summary analysis and this conservative approach may have contributed to underestimating the association as calculated in the previous meta-analysis (31). Summary and pooled AORs were represented as point estimates and 95% CIs on a forest plot. Heterogeneity in the included studies was assessed using the Q test and quantified using *I^2^*. *I^2^* values below 25% were considered as homogeneity; 25% to <50%, low heterogeneity; 50% to <75%, moderate heterogeneity; and at least 75%, substantial heterogeneity (33). Egger’s and/or Begg’s tests were used to evaluate publication bias, and funnel plots were performed to visually assess publication bias of included studies (34). Sensitivity analyses were used to assess the overall robustness of the included studies. Quality and risk of bias were assessed for each study using the Newcastle-Ottawa Scale (NOS) or a modified NOS (35).

## Results

### Characteristics of the studies

**Figure 1:**
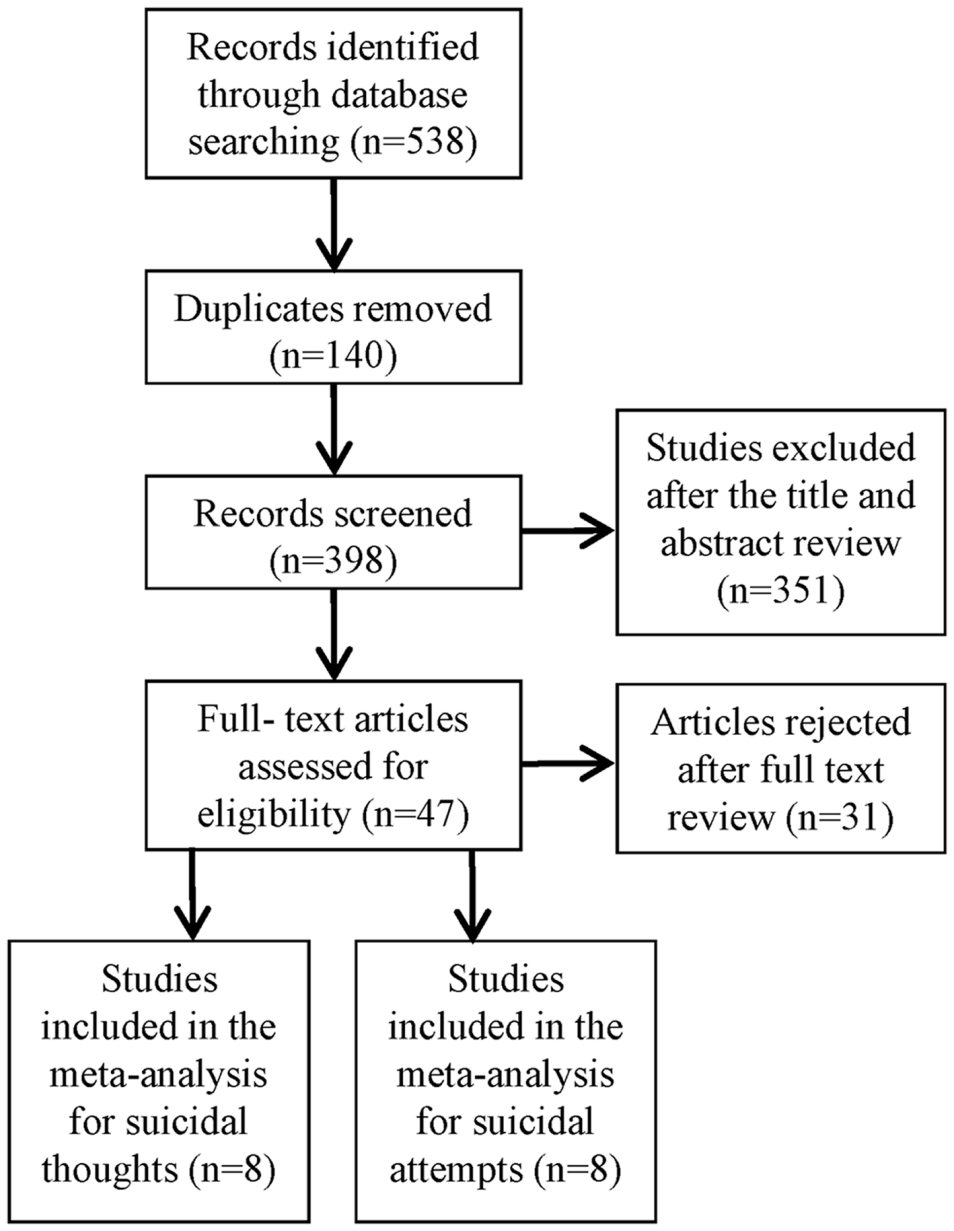
Flowchart of identification and selection of studies included in the meta-analysis.

A total of 538 potentially eligible articles were identified after searching the three databases and removing duplicates. After eliminating 351 articles based on the title and abstract review, the remaining 47 were read in full and 16 were ultimately included in the meta-analysis (Figure 1). The details of the included study are in Table 1 and Table 2.

**Table 1:** Description of studies about suicidal ideation and migraine included in the meta-analysis.

Author (year)	Study design	Sample size	Age (years)	Criteria used to assess suicidal ideation	Criteria used to assess migraine	Stratification by migraine subtype	Unadjusted OR (95% CI)	Adjusted OR (95% CI)	Adjustment variables
Berhane (2018)	Cross-sectional	1,060	35.7 ± 12.1	During that period, did you ever think that it would be better if you were dead	ICHD-II criteria	No	2.91 (2.06–4.12)	1.49 (0.93–2.39)	Age, sex, education, BMI categories, past year alcohol consumption, and lifetime depression
Campbell (2015)	Cross-sectional	8,841	16–85	Serious thoughts about committing suicide	Self-reported by the respondent based on the WMH-CIDI chronic conditions module	No	2.55 (2.02–3.19)	1.49 (1.12–2.00)	Socio-demographic factors, mental health and a number of other pain conditions (i.e., arthritis, chronic migraines, chronic back or neck problems)
Kim (2014)	Cross-sectional	238	Mean 39.1	Defined using the SSI-Beck score, >8 for adults, >13 for college students, and > 15 for high school students	Revised ICHD-II criteria	No	5.09 (1.17–22.10)	1.51 (0.31–7.50)	Age, sex, BDI and BAI
Fuller-Thomson (2013)	Cross-sectional	5,788	15–19	Ask “have you ever seriously considered committing suicide or taking your own life?”	Self-reported physician diagnosed migraine headache	No	NA	1.25 (0.64–2.45)	Gender, school attendance, race, living arrangement, lack of food, lack of money, smoking, alcohol dependency, self-perceived health status, activities
Wang (2009)	Cross-sectional	3,963	13–15	Adolescent Depression Inventory (Item17)	Structured questionnaire that uses IHS criteria (2004)	Yes	2.9 (2.3–3.6)	MA: 1.79 (1.07–2.99); MO: 1.04 (0.77–1.40)	Age, gender, depression, living arrangements
Ratcliffe (2008)	Cross-sectional	36,984	>15	Asked if they had ever seriously thought about committing suicide or taking their own lives	Self-reported physician diagnosed migraine headache	No	NA	1.35 (1.04–1.75)	Sex, age, marital status, education, comorbidity (≥mental disorders), other chronic pain disorders (arthritis, back problems, and migraine)
Wang (2007)	Cross-sectional	121	Mena 13.8	Mini-International Neuropsychiatric Interview-Kid for children and adolescents suicidality module	A structured questionnaire that uses IHS criteria (2006)	Yes	4.3 (1.2–15.5)	MA: 7.8 (1.4–44.6); MO: 2.1 (0.5–8.5)	Age, gender, major depression, anxiety disorders
Breslau (1992)	Cross-sectional	1,007	21–30	Ask “Have you ever felt so low you thought about committing suicide?”	A structured questionnaire that uses IHS criteria (1988)	Yes	NA	MA: 2.4 (1.1–5.3); MO: 1.7 (0.8–3.4)	Coexisting mania, dysthymia, anxiety disorders, substance use disorders

OR, Odds Ratio; CI, Confidence Interval; SSI, Scale for Suicidal Ideation; BMI, Body Mass Index; BDI, Beck Depression Inventory; BAI, Beck Anxiety Inventory; SA, status migraine; RA, regular migraine; MA, migraine with aura,; MO, migraine without aura; ICHD, The international classification of headache disorders; IHS, The International Headache Society; WMH-CIDI, World Mental Health Survey Initiative version of the Composite International Diagnostic Interview; ICD, The international classification disease.

**Table 2:** Description of studies about suicidal attempts and migraine included in the meta-analysis.

Author (year)	Country	Study design	Sample size	Age (years)	Criteria used to assess suicidal attempts	Criteria used to assess migraine	Stratification by migraine subtype	Unadjusted OR (95% CI)	Adjusted OR (95% CI)	Adjustment variables
Fuller-Thomson (2019)	Canada	Cross-sectional	21,774	>18	Attempted suicide or tried to take (their) own life	Self-reported physician diagnosed migraine headache	Yes	NA	1.77 (1.44–2.18)	Demographics, socio-economic status, three adverse childhood experiences, history of substance abuse, history of mental illness, and pain
Harnod (2018)	Taiwan	Cross-sectional	175,307	>20	ICD-9-CM codes E950-E959	ICD-9-CM code 346 except 346.9 or ICD-9-CM code 346.9, and without code 346.90 or 346.91	Yes	SA: 1.82 (1.15–2.90); RA: 0.35 (0.91–2.02)	SA: 1.81 (1.14–2.89); RA: 1.34 (0.90–2.00)	Age, sex, monthly income, urbanization level, occupation, and comorbidities
Berhane (2018)	America	Cross-sectional	1,060	35.7 ± 12.1	Did you make a suicide attempt	ICHD-II criteria	No	2.91 (2.06–4.12)	1.49 (0.93–2.39)	Age, sex, education, BMI categories, past year alcohol consumption, and lifetime depression
Calati (2017)	France	Cohort study	1965	>65	Diagnostic and Statistical Manual of Mental Disorders	International Headache Society (IHS)	No	NA	1.92 (1.17–3.15)	Gender, living alone, tobacco and alcohol consumption, depressive, manic/hypomanic and anxiety disorders
Campbell (2015)	Australian	Cross-sectional	8,841	16–85	Adopted from the WMH-CIDI without modification	Self-reported by the respondent based on the WMH-CIDI chronic conditions module	No	2.65 (1.84–3.79)	1.31 (0.82–2.08)	Socio-demographic factors, mental health and a number of other pain conditions (i.e., arthritis, chronic migraines, chronic back or neck problems)
Breslau (2012)	America	Cohort study	1,186	40.5 ± 8.6	Did you attempt suicide	first and second editions of the ICHD	No	7.21(3.21–16.2)	4.43(1.93–10.2)	Sex, major depression, anxiety, baseline alcohol or drug use disorder
Ratcliffe (2008)	Canada	Cross-sectional	36,984	≥15	Asked if they had attempted suicide or tried to take their own lives	Self-reported physician diagnosed migraine headache	No	NA	2.05 (1.21–3.45)	Sex, age, marital status, education, comorbidity (3 or more mental disorders), and other chronic pain disorders (arthritis, back problems, and migraine)
Breslau (1991)	America	Cross-sectional	1,007	21–30	Diagnostic Interview Schedule	A structured questionnaire that uses IHS criteria (1988)	Yes	NA	MA: 2.99 (1.35–6.61); MO: 1.59 (0.63–4.01)	Sex, major depression, affective disorders, anxiety disorders, substance use disorders

OR, Odds Ratio; CI, Confidence Interval; SSI, Scale for Suicidal Ideation; BMI, Body Mass Index; BDI, Beck Depression Inventory; BAI, Beck Anxiety Inventory; SA, status migraine, RA, regular migraine; MA, migraine with aura, MO, migraine without aura; ICHD, The international classification of headache disorders; IHS, The International Headache Society; WMH-CIDI, World Mental Health Survey Initiative version of the Composite International Diagnostic Interview; ICD, The international classification disease.

### Overall AOR for suicide ideation in migraineurs

**Figure 2:**
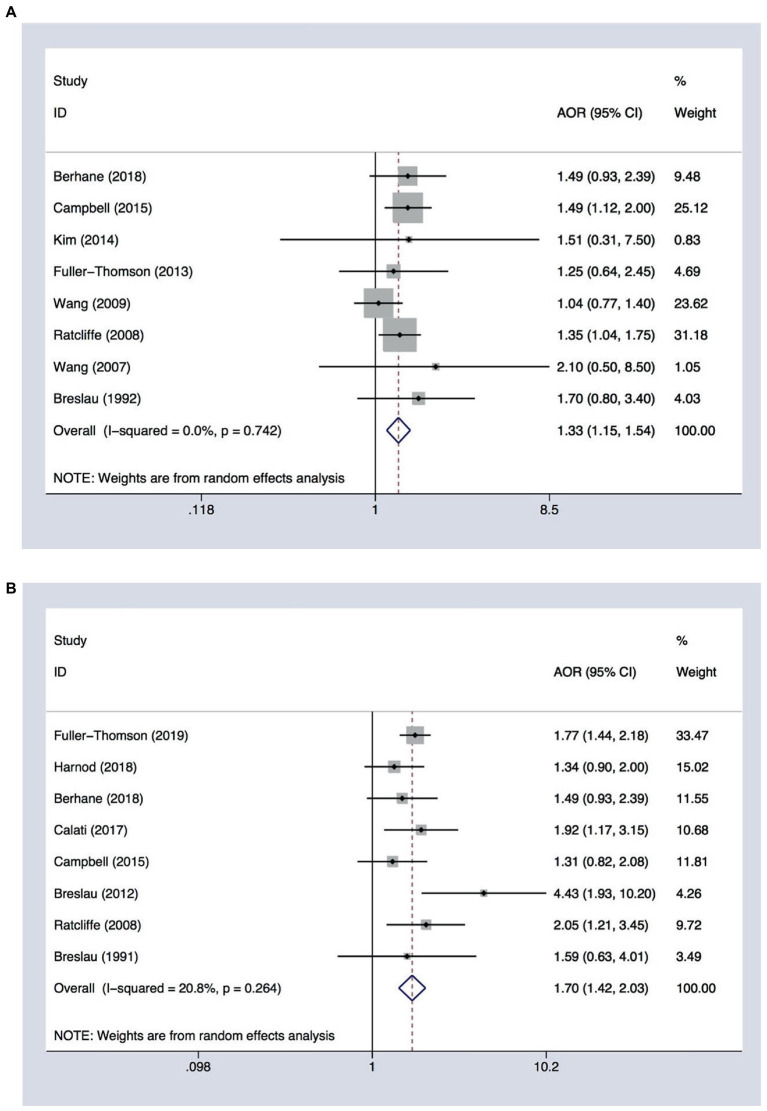
Forest plot assessing the association between migraine and **(A)** suicide ideation (*n* = 8) or **(B)** suicide attempts (*n* = 8). AOR, adjusted odds ratio; CI, confidence interval.

Eight cross-sectional studies evaluating suicide ideation in patients with migraine, which comprised a total of 58,002 participants, were included in the meta-analysis (Table 1). The unadjusted OR for suicide ideation in migraineurs ranged from 2.55 (95% CI 2.02–3.19) in Australia to 5.09 (95% CI 1.17–22.10) in South Korea, while the AOR varied from 1.25 (95% CI 0.64–2.45) in Canada to 1.49 (95% CI 1.12–2.00) in Australia. According to migraine subtypes, we observed that the risk of suicide ideation was higher in patients suffering from migraine with aura than in those with migraine without aura. The AOR ranged from 1.79 (95% CI 1.07–2.99) to 7.8 (95% CI 1.44–4.6) in migraine with aura, and from 1.04 (95% CI 0.77–1.40) to 2.1 (95% CI 0.5–8.5) in migraine without aura. In the meta-analysis, we used the pooled AOR of migraine without aura to calculate the overall pooled AOR. Random-effects meta-analysis of pooled data showed that patients with migraine were at higher risk of suicide ideation (AOR 1.33, 95% CI 1.15–1.54). Our analysis indicated homogeneity among the studies (*I^2^* = 0%, *p* = 0.742; Figure 2A).

### Overall AOR for suicide attempts in migraineurs

Eight studies analyzing suicide attempts in migraine, including six cross-sectional studies and two cohort studies involving 248,124 participants, were included in the meta-analysis. The unadjusted OR for suicide attempts in migraineurs ranged from 1.35 (95% CI 0.91–2.02) in Taiwan to 7.21 (95% CI 3.21–16.2) in the United States, while the AOR ranged from 1.31 (95% CI 0.82–2.08) in Australia to 4.43 (95% CI 1.93–10.2) in the United States. Random-effect meta-analysis showed that patients with migraine were at higher risk of suicide attempts (AOR 1.70, 95% CI 1.42–2.03). Our analysis showed the homogeneity of the included studies (*I^2^* = 20.8%, *p* = 0.264; Figure 2B).

### Age subgroup analysis in migraine with suicide ideation

**Figure 3:**
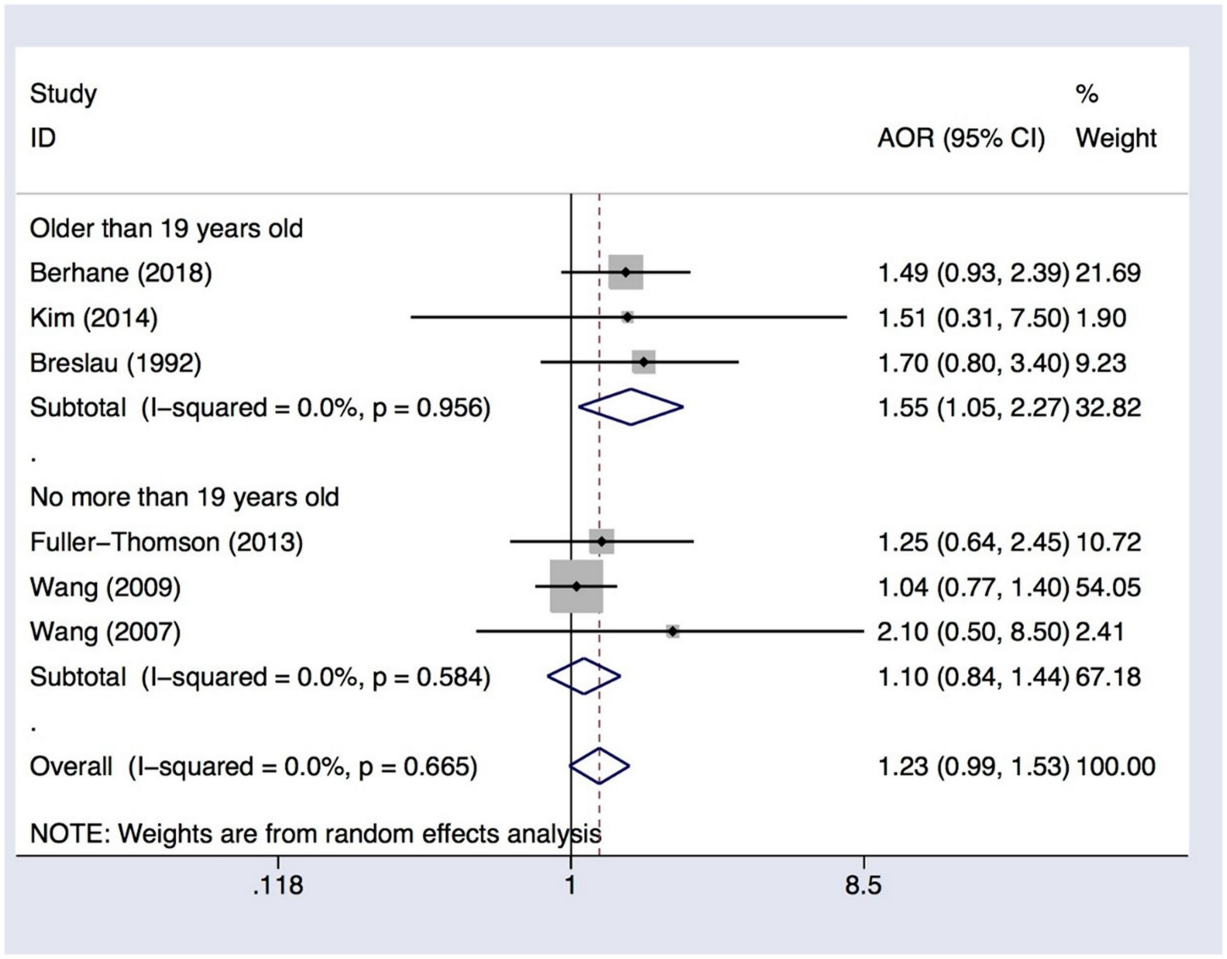
Subgroup analysis of the association between migraine and suicide ideation among subjects no more than or older than 19 years. AOR, adjusted odds ratio; CI, confidence interval.

We conducted a subgroup analysis in migraine with suicide ideation to address potential differences between adolescents (no more than 19 years) and adults (over 19 years of age). In the subgroup analysis, the AOR in migraine without aura was used to calculate the overall pooled AOR. The pooled AOR in adolescents was 1.10 (95% CI 0.84–1.44, I-squared = 0.0%, *p* = 0.584), indicating no significant association between migraine and suicide ideation (Figure 3). In adults, the pooled AOR was 1.55 (95% CI 1.05–2.27, *I^2^* = 0.0%, *p* = 0.956), confirming that adults with migraines were at higher risk of suicide ideation (Figure 3).

### Sensitivity analysis

To test the robustness of our findings, we performed a sensitivity analysis by omitting individual studies one at a time. We discovered that removing each study sequentially had no effect on the results of primary overall analyses (Supplementary Figure 1).

### Publication bias

**Figure 4:**
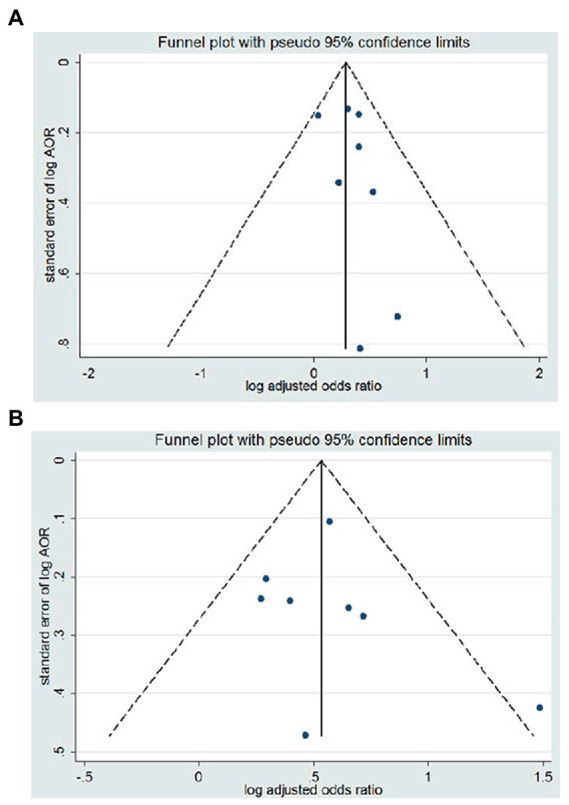
Funnel plots of the article included the association between migraine and **(A)** suicide ideation or **(B)** suicide attempts. AOR, adjusted odds ratio; CI, confidence interval.

Visual inspection of the associated Begg’s funnel plot showed evidence of a moderate publication bias (Figure 4). However, Begg’s tests showed *p*-value was 0.902 and 0.373 in the risk analysis of suicide ideation and suicide attempts, respectively. Egger’s test showed that the *p* values are 0.108 and 0.619, respectively. Our study suggesting no significant risk of publication bias.

### Quality assessment

Each study was given a NOS quality score (36). Quality scores for each study ranged from 5/7 to 7/7, representing a quality assessment between “good” and “fair” quality scores (Supplementary Tables 2, 3).

## Discussion

As far as we know, our meta-analysis examines the association between migraine and suicide attempts. A previous meta-analysis indicated that migraineurs are at higher risk of suicide ideation (31). Our meta-analysis, which included three new studies and excluded one study concerning that previous work, confirmed the association between migraine and suicide ideation (AOR 1.33, 95% CI 1.15–1.54). Similarly, our results revealed a significant association between migraine and suicide attempts (AOR 1.70, 95% CI 1.48–1.97). Depression, anxiety, sleep disorders, post-traumatic stress disorder, and bipolar spectrum disorder are usual comorbidities of migraine (37), and they have shown a strong association with increased risk of suicide ideation or attempts (38–42). This was further supported by our conclusions, which confirmed that migraineurs were at higher risk of suicide ideation/attempts.

The mechanisms behind our findings can be explained in the following ways. First, there may be an association between migraine/suicide ideation/suicide attempts and serotonin levels, and abnormalities and deficiencies in serotonin transporters, receptors, and metabolites are associated with migraine and suicide ideation/attempts (43). Second, there may be common neurobiological and neuroanatomical mechanisms underlying migraine and suicidal behavior, and studies have shown that changes in gray matter volume in the dorsolateral prefrontal cortex are independently associated with the occurrence of migraine and suicidal behavior (44). Third, dysfunction of the hypothalamic–pituitary–adrenal axis may be associated with migraine and suicidal behavior (45). Fourth, concomitant psychiatric co-morbidities in migraineurs, such as depression and anxiety, also increase the risk of suicide (46). Fifth, migraine attacks limit patients’ daily activities, which may also be an important factor contributing to suicide. However, more research is needed in the future to explore the possible mechanisms underlying the association between migraine and suicide behavior, as these may be key factors in preventing suicide and improving the quality of life of migraineurs.

In the age subgroup analysis, although the risk of migraine and suicidal ideation in adolescents younger than 19 years was not statistically significant. We can preliminarily observe a trend toward higher risk of suicidal ideation in adult migraineurs than in adolescent migraineurs, which is consistent with previous observational findings that children and adolescents under 15 years of age present the lowest global suicide rate, which steadily increases over 70 years of age (47). In addition, the lack of increased risk of suicidal ideation in migraineurs no older than 19 years may also be related to the small sample size of migraineurs younger than 19 years included in the study. Therefore, further research is needed to examine the relationship between migraine and suicidal ideation/attempt and to explore the mechanisms behind them, especially in adolescents.

Our study presents several limitations. First, the number of available studies on adolescents was small. Second, most of the publications in the meta-analysis were cross-sectional, and therefore more prospective cohort studies are urgently needed. Third, although we performed subgroup analysis and showed stable results. Our research will still be affected by some untreated confounding factors. Fourth, although Begg’s and Egger’s tests showed no significant publication bias, potential publication bias could not be completely ruled out. Finally, our results of the present meta-analysis should be confirmed and extended in larger studies considering potential ethnic and geographic differences.

## Conclusion

The present meta-analysis identified a significant association between migraine and suicide ideation and attempts, although potential ethnic and geographic differences were not evaluated. The available evidence suggests that psychological interventions should be acknowledged as a fundamental component of migraine patient care.

## Data availability statement

The original contributions presented in the study are included in the article/Supplementary material, further inquiries can be directed to the corresponding author.

## Author contributions

HW and JR: conceptualization. HW, YL, HL, and JR: methodology, formal analysis and investigation. HW: writing–original draft preparation. JR: writing–review and editing and supervision. All authors contributed to the article and approved the submitted version.

## Conflict of interest

The authors declare that the research was conducted in the absence of any commercial or financial relationships that could be construed as a potential conflict of interest.

## Publisher’s note

All claims expressed in this article are solely those of the authors and do not necessarily represent those of their affiliated organizations, or those of the publisher, the editors and the reviewers. Any product that may be evaluated in this article, or claim that may be made by its manufacturer, is not guaranteed or endorsed by the publisher.

## Abbreviations

AOR: adjusted odds ratios
CIs: confidence intervals
PRISMA: Preferred Reporting Items for Systematic Review and Meta-Analyses.

## References

[1] Burch RC Loder S Loder E Smitherman TA. The prevalence and burden of migraine and severe headache in the United States: updated statistics from government health surveillance studies. Headache. (2015) 55:21–34. doi: 10.1111/head.12482, PMID: 25600719

[2] Steiner TJ Stovner LJ Vos T. GBD 2015: migraine is the third cause of disability in under 50s. J Headache Pain. (2016) 17:104. doi: 10.1186/s10194-016-0699-5, PMID: 27844455 PMC5108738

[3] Smitherman TA Burch R Sheikh H Loder E. The prevalence, impact, and treatment of migraine and severe headaches in the United States: a review of statistics from national surveillance studies. Headache. (2013) 53:427–36. doi: 10.1111/head.12074, PMID: 23470015

[4] Lipton RB Hamelsky SW Kolodner KB Steiner TJ Stewart WF. Migraine, quality of life, and depression: a population-based case-control study. Neurology. (2000) 55:629–35. doi: 10.1212/WNL.55.5.629, PMID: 10980724

[5] Linde M Dahlof C. Attitudes and burden of disease among self-considered Migraineurs — a nation-wide population-based survey in Sweden. Cephalalgia. (2004) 24:455–65. doi: 10.1111/j.1468-2982.2004.00703.x, PMID: 15154855

[6] Lipton RB Bigal ME Kolodner K Stewart WF Liberman JN Steiner TJ. The family impact of migraine: population-based studies in the USA and UK. Cephalalgia. (2003) 23:429–40. doi: 10.1046/j.1468-2982.2003.00543.x, PMID: 12807522

[7] Marcus DA Bhowmick A. Fibromyalgia comorbidity in a community sample of adults with migraine. Clin Rheumatol. (2013) 32:1553–6. doi: 10.1007/s10067-013-2310-7, PMID: 23743661

[8] Nye BL Thadani VM. Migraine and epilepsy: review of the literature. Headache. (2015) 55:359–80. doi: 10.1111/head.12536 25754865

[9] Harriott AM Barrett KM. Dissecting the association between migraine and stroke. Curr Neurol Neurosci Rep. (2015) 15:5. doi: 10.1007/s11910-015-0530-8 25652090

[10] Peterlin BL Nijjar SS Tietjen GE. Post-traumatic stress disorder and migraine: epidemiology, sex differences, and potential mechanisms. Headache. (2011) 51:860–8. doi: 10.1111/j.1526-4610.2011.01907.x, PMID: 21592096 PMC3974501

[11] Nock MK Borges G Bromet EJ Alonso J Angermeyer M Beautrais A. Cross-national prevalence and risk factors for suicidal ideation, plans and attempts. Br J Psychiatry. (2008) 192:98–105. doi: 10.1192/bjp.bp.107.040113, PMID: 18245022 PMC2259024

[12] Bourke SC Tomlinson M Williams TL Bullock RE Shaw PJ Gibson GJ. Effects of non-invasive ventilation on survival and quality of life in patients with amyotrophic lateral sclerosis: a randomised controlled trial. Lancet Neurol. (2006) 5:140–7. doi: 10.1016/S1474-4422(05)70326-4, PMID: 16426990

[13] Nock MK Borges G Bromet EJ Cha CB Kessler RC Lee S. Suicide and suicidal behavior. Epidemiol Rev. (2008) 30:133–54. doi: 10.1093/epirev/mxn002, PMID: 18653727 PMC2576496

[14] Karbeyaz K Toygar M Celikel A. Completed suicide among university student in Eskisehir, Turkey. J Forensic Leg Med. (2016) 44:111–5. doi: 10.1016/j.jflm.2016.09.010, PMID: 27744134

[15] Klonsky ED May AM Saffer BY. Suicide, suicide attempts, and suicidal ideation. Annu Rev Clin Psychol. (2016) 12:307–30. doi: 10.1146/annurev-clinpsy-021815-093204, PMID: 26772209

[16] Colman I Kingsbury M Sareen J Bolton J van Walraven C. Migraine headache and risk of self-harm and suicide: a population-based study in Ontario, Canada. Headache. (2016) 56:132–40. doi: 10.1111/head.12710, PMID: 26518353

[17] Campbell G Darke S Bruno R Degenhardt L. The prevalence and correlates of chronic pain and suicidality in a nationally representative sample. Aust N Z J Psychiatry. (2015) 49:803–11. doi: 10.1177/0004867415569795, PMID: 25698809

[18] Wang SJ Fuh JL Juang KD Lu SR. Migraine and suicidal ideation in adolescents aged 13 to 15 years. Neurology. (2009) 72:1146–52. doi: 10.1212/01.wnl.0000345362.91734.b3, PMID: 19332691

[19] Ratcliffe GE Enns MW Belik SL Sareen J. Chronic pain conditions and suicidal ideation and suicide attempts: an epidemiologic perspective. Clin J Pain. (2008) 24:204–10. doi: 10.1097/AJP.0b013e31815ca2a3, PMID: 18287825

[20] Wang SJ Juang KD Fuh JL Lu SR. Psychiatric comorbidity and suicide risk in adolescents with chronic daily headache. Neurology. (2007) 68:1468–73. doi: 10.1212/01.wnl.0000260607.90634.d6, PMID: 17470748

[21] Breslau N. Migraine, suicidal ideation, and suicide attempts. Neurology. (1992) 42:392–5. 1736172 10.1212/wnl.42.2.392

[22] Fuller-Thomson E Hodgins GA. Suicide attempts among those with migraine: findings from a nationally representative Canadian study. Arch Suicide Res. (2020) 24:360–79. doi: 10.1080/13811118.2019.1578710, PMID: 30945611

[23] Calati R Courtet P Norton J Ritchie K Artero S. Association between lifetime headache and history of suicide attempts in the elderly. Eur Psychiatry. (2017) 41:132–9. doi: 10.1016/j.eurpsy.2016.10.009, PMID: 28152434

[24] Breslau N Schultz L Lipton R Peterson E Welch KMA. Migraine headaches and suicide attempt. Headache. (2012) 52:723–31. doi: 10.1111/j.1526-4610.2012.02117.x 22404176

[25] Berhane HY Jamerson-Dowlen B Friedman LE Berhane Y Williams MA Gelaye B. Association between migraine and suicidal behavior among Ethiopian adults. BMC Psychiatry. (2018) 18:46. doi: 10.1186/s12888-018-1629-7, PMID: 29433452 PMC5809936

[26] Kim SY Park SP. Suicidal ideation and risk factors in Korean migraine patients. J Clin Neurosci. (2014) 21:1699–704. doi: 10.1016/j.jocn.2014.03.016, PMID: 24998861

[27] Fuller-Thomson E Schrumm M Brennenstuhl S. Migraine and despair: factors associated with depression and suicidal ideation among Canadian Migraineurs in a population-based study. Depress Res Treat. (2013) 2013:401487. doi: 10.1155/2013/401487 24224086 PMC3810321

[28] Harnod T Lin CL Kao CH. Risk and predisposing factors for suicide attempts in patients with migraine and status Migrainosus: a Nationwide population-based study. J Clin Med. (2018) 7:269. doi: 10.3390/jcm7090269, PMID: 30208570 PMC6162830

[29] Breslau N Davis GC Andreski P. Migraine, psychiatric disorders, and suicide attempts: an epidemiologic study of young adults. Psychiatry Res. (1991) 37:11–23. doi: 10.1016/0165-1781(91)90102-U, PMID: 1862159

[30] Pei JH Wang XL Yu Y Zhang YB Gou L Nan RL. Prevalence of suicidal ideation and suicide attempt in patients with migraine: a systematic review and meta-analysis. J Affect Disord. (2020) 277:253–9. doi: 10.1016/j.jad.2020.08.019, PMID: 32841826

[31] Friedman LE Gelaye B Bain PA Williams MA. A systematic review and meta-analysis of migraine and suicidal ideation. Clin J Pain. (2017) 33:659–65. doi: 10.1097/AJP.0000000000000440, PMID: 27648590 PMC5357206

[32] Moher D Liberati A Tetzlaff J Altman DG. Preferred reporting items for systematic reviews and meta-analyses: the PRISMA statement. Int J Surg. (2010) 8:336–41. doi: 10.1016/j.ijsu.2010.02.007 20171303

[33] Higgins JP Thompson SG Deeks JJ Altman DG. Measuring inconsistency in meta-analyses. BMJ. (2003) 327:557–60. doi: 10.1136/bmj.327.7414.557, PMID: 12958120 PMC192859

[34] Egger M Smith GD Phillips AN. Meta-analysis: principles and procedures. BMJ. (1997) 315:1533–7. 9432252 10.1136/bmj.315.7121.1533 PMC2127925

[35] Fralick M Sy E Hassan A Burke MJ Mostofsky E Karsies T. Association of Concussion with the risk of suicide: a systematic review and meta-analysis. JAMA Neurol. (2019) 76:144–51. doi: 10.1001/jamaneurol.2018.3487, PMID: 30419085 PMC6439954

[36] Stang A. Critical evaluation of the Newcastle-Ottawa scale for the assessment of the quality of nonrandomized studies in meta-analyses. Eur J Epidemiol. (2010) 25:603–5. doi: 10.1007/s10654-010-9491-z, PMID: 20652370

[37] Minen MT Begasse de Dhaem O Kroon van Diest A Powers S Schwedt TJ Lipton R. Migraine and its psychiatric comorbidities. J Neurol Neurosurg Psychiatry. (2016) 87:741–9. doi: 10.1136/jnnp-2015-312233 26733600

[38] Bernert RA Nadorff MR. Sleep disturbances and suicide risk. Sleep Med Clin. (2015) 10:35–9. doi: 10.1016/j.jsmc.2014.11.004 26055671

[39] Stanley IH Boffa JW Rogers ML Hom MA Albanese BJ Chu C. Anxiety sensitivity and suicidal ideation/suicide risk: a meta-analysis. J Consult Clin Psychol. (2018) 86:946–60. doi: 10.1037/ccp0000342, PMID: 30335426 PMC6469498

[40] Ribeiro JD Huang X Fox KR Franklin JC. Depression and hopelessness as risk factors for suicide ideation, attempts and death: meta-analysis of longitudinal studies. Br J Psychiatry. (2018) 212:279–86. doi: 10.1192/bjp.2018.27, PMID: 29587888

[41] Schaffer A Isometsä ET Tondo L H Moreno D Turecki G Reis C. International Society for Bipolar Disorders Task Force on suicide: meta-analyses and meta-regression of correlates of suicide attempts and suicide deaths in bipolar disorder. Bipolar Disord. (2015) 17:1–16. doi: 10.1111/bdi.12271, PMID: 25329791 PMC6296224

[42] Krysinska K Lester D. Post-traumatic stress disorder and suicide risk: a systematic review. Arch Suicide Res. (2010) 14:1–23. doi: 10.1080/13811110903478997, PMID: 20112140

[43] Panconesi A. Serotonin and migraine: a reconsideration of the central theory. J Headache Pain. (2008) 9:267–76. doi: 10.1007/s10194-008-0058-2, PMID: 18668197 PMC3452194

[44] Chen WT Chou KH Lee PL Hsiao FJ Niddam DM Lai KL. Comparison of gray matter volume between migraine and "strict-criteria" tension-type headache. J Headache Pain. (2018) 19:4. doi: 10.1186/s10194-018-0834-6, PMID: 29335889 PMC5768588

[45] Peres MF Sanchez del Rio M Seabra ML Tufik S Abucham J Cipolla-Neto J. Hypothalamic involvement in chronic migraine. J Neurol Neurosurg Psychiatry. (2001) 71:747–51. doi: 10.1136/jnnp.71.6.747, PMID: 11723194 PMC1737637

[46] Antonaci F Nappi G Galli F Manzoni GC Calabresi P Costa A. Migraine and psychiatric comorbidity: a review of clinical findings. J Headache Pain. (2011) 12:115–25. doi: 10.1007/s10194-010-0282-4, PMID: 21210177 PMC3072482

[47] Bachmann S. Epidemiology of suicide and the psychiatric perspective. Int J Environ Res Public Health. (2018) 15:1425. doi: 10.3390/ijerph15071425, PMID: 29986446 PMC6068947

