# Supplementary material for: Associations of migraines with suicide ideation or attempts: A meta-analysis
Source: Front Public Health. 2023 Mar 24;11:1140682. doi: 10.3389/fpubh.2023.1140682 (PMC10080086; doi:10.3389/fpubh.2023.1140682)

Supplementary Fig. 1 (a) Sensitivity analysis of the association between migraine and suicide ideation. (b) Sensitivity analysis of the association between migraine and suicide attempts. CI, confidence interval.

(a)


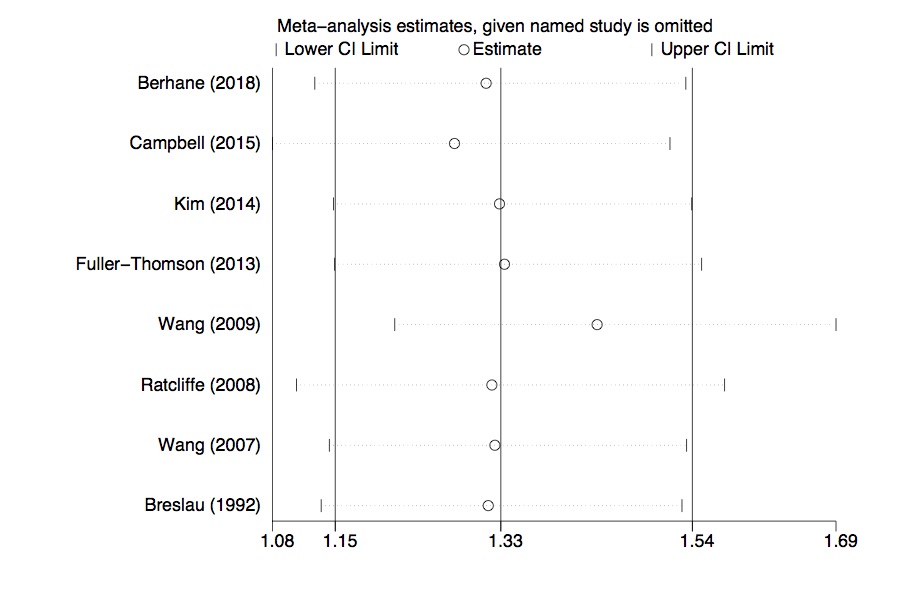


(b)


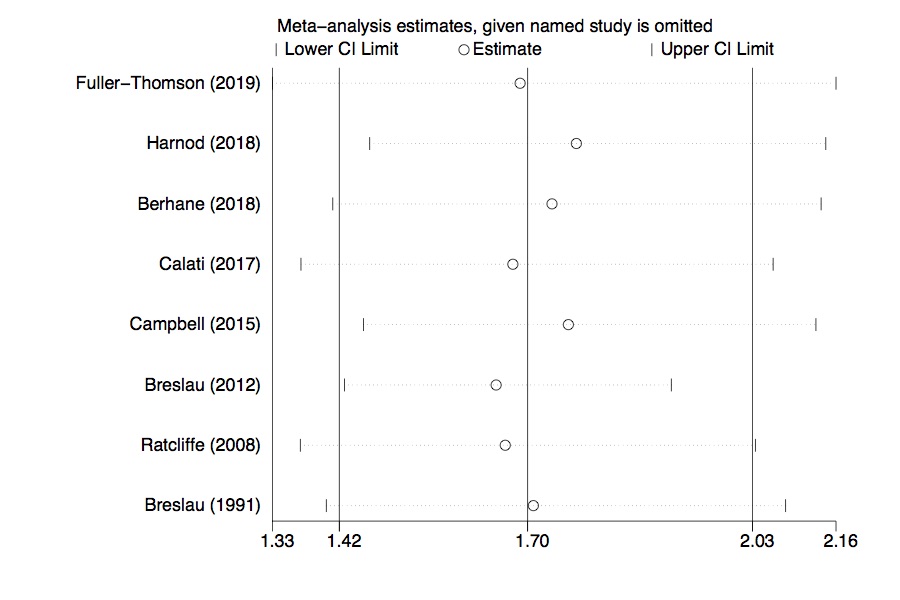

Supplement: Supplementary file 1 [file Data_Sheet_1.DOCX]
