# Supplementary material for: Associations of migraines with suicide ideation or attempts: A meta-analysis
Source: Front Public Health. 2023 Mar 24;11:1140682. doi: 10.3389/fpubh.2023.1140682 (PMC10080086; doi:10.3389/fpubh.2023.1140682)
Supplement: Supplementary file 2 [file Data_Sheet_2.PDF]

International Platform of Registered Systematic Review and Meta-analysis Protocols

INPLASY PROTOCOL

To cite: Wei et al. Associations of migraines with suicide ideation or attempts: a meta-analysis. Inplasy protocol 202330019. doi: 10.37766/inplasy2023.3.0019

Received: 06 March 2023

Published: 06 March 2023

Corresponding author:
Junwei Ren

renjunweia2008@163.com

Author Affiliation:
Chongqing University FuLing Hospital.

Support: **Not available.**

Review Stage at time of this submission: **Completed but not published.**

Conflicts of interest:
None declared.

Associations of migraines with suicide ideation or attempts: a meta-analysis

Wei, HJ[1]; Li, Y[2]; Lei, H[3]; Ren, JW[4].

Review question / Objective: Associations of migraines with suicide ideation or attempts.
Condition being studied: Migraines, the second most common primary headache, are recurrent disabling headaches associated with neurologic symptoms, including severe throbbing head pain, with or without visual disturbances. According to the Global Burden of Disease Study, migraine is the third leading cause of disability in people under 50 years, and it is the most common neurological disorder, with an estimated global prevalence ranging from 11% to 23%. Sufferers of migraine often have a lower health-related quality of life than non-migraineurs, as well as influence work productivity and in social and family relationships. Migraines are known to be comorbid with other neurological and psychiatric disorders including fibromyalgia, epilepsy, stroke, multiple sclerosis, panic disorder, depression, and post-traumatic stress disorder.

INPLASY registration number: This protocol was registered with the International Platform of Registered Systematic Review and Meta-Analysis Protocols (INPLASY) on 06 March 2023 and was last updated on 06 March 2023 (registration number INPLASY202330019).

INTRODUCTION

Review question / Objective: Associations of migraines with suicide ideation or attempts.

Condition being studied: Migraines, the second most common primary headache, are recurrent disabling headaches associated with neurologic symptoms, including severe throbbing head pain, with or without visual disturbances. According to the Global Burden of Disease Study, migraine is the third leading cause of disability in people under 50 years, and it is the most common neurological disorder, with an estimated global prevalence ranging from 11% to 23%. Sufferers of

Wei et al. Inplasy protocol 202330019. doi:10.37766/inplasy2023.3.0019 Downloaded from https://inplasy.com/inplasy-2023-3-0019/

migraine often have a lower health-related quality of life than non-migraineurs, as well as influence work productivity and in social and family relationships. Migraines are known to be comorbid with other neurological and psychiatric disorders including fibromyalgia, epilepsy, stroke, multiple sclerosis, panic disorder, depression, and post-traumatic stress disorder.

METHODS

Participant or population: Migraines.

Intervention: Assessment of suicidal ideation or attempt.

Comparator: Suicide ideation or attempts.

Study designs to be included: Meta analysis.

Eligibility criteria: Studies were included if they fulfilled the following requirements: (a) they were observational studies, including cohort, case-control, or cross-sectional studies, evaluating associations of migraines with suicide ideation or attempts; (b) they reported adjusted odds ratios (AORs) with 95% confidence intervals (CIs) in the migraine cohort, and compared the results with a non-migraineur population, and (c) they were published in English. Exclusion criteria during the abstract review included: (a) articles that were meeting abstracts, case reports, discussions, editorials, reviews, letters, or commentaries; or (b) articles analyzing migraine in pregnant women.

Information sources: PubMed, EMBASE, and Web of Science databases.

Main outcome(s): Suicide ideation or attempts.

Quality assessment / Risk of bias analysis: Each study was given a Newcastle-Ottawa Scale quality score.

Strategy of data synthesis: We used Stata version 12.0 (StataCorp, College Station, TX, USA) statistical software to generate summary statistics and pooled AORs using a random model.

Subgroup analysis: We conducted a subgroup analysis in migraine with suicide ideation to address potential differences between adolescents (no more than 19 years) and adults (over 19 years of age).

Sensitivity analysis: We performed a sensitivity analysis by omitting the individual studies one at a time to test the robustness of our findings.

Country(ies) involved: China.

Keywords: migraine, suicide ideation, suicide attempt, meta-analysis.

Contributions of each author:
Author 1 - Huijie Wei.
Author 2 - Yu Li.
Author 3 - Hua Lei.
Author 4 - Junwei Ren.

Wei et al. Inplasy protocol 202330019. doi:10.37766/inplasy2023.3.0019 Downloaded from https://inplasy.com/inplasy-2023-3-0019/
