# Supplementary material for: Associations of migraines with suicide ideation or attempts: A meta-analysis
Source: Front Public Health. 2023 Mar 24;11:1140682. doi: 10.3389/fpubh.2023.1140682 (PMC10080086; doi:10.3389/fpubh.2023.1140682)
Supplement: Supplementary file 3 [file Table_1.DOCX]

**Search Strategy**

PubMed：

((migraine[Title/Abstract]) OR (headache[Title/Abstract])) AND ((suicide[Title/Abstract]) OR (suicides[Title/Abstract]))

Embase:

((migraine or headache) and suicide).af.

((migraine or headache) and suicides).af.

Web of Science:

(migraine OR headache) AND (suicide or suicides)
