# Supplementary material for: Associations of migraines with suicide ideation or attempts: A meta-analysis
Source: Front Public Health. 2023 Mar 24;11:1140682. doi: 10.3389/fpubh.2023.1140682 (PMC10080086; doi:10.3389/fpubh.2023.1140682)
Supplement: Supplementary file 5 [file Table_3.DOCX]

| **Supplementary Table 2. Risk of bias of the included studies** | | | | | | | | | | |  |  |
| --- | --- | --- | --- | --- | --- | --- | --- | --- | --- | --- | --- | --- |
| **Author** | **Study design** | **Selection** | | | | **Comparability** | **Outcome/Esposure** | | | **Score** | **Rank** |  |
|  |  | **1** | **2** | **3** | **4** | **5** | **6** | **7** | **8** |  |  |  |
| Berhane | Cross-sectional | ★ |  | ★ | ★ | ★ ★ | ★ | ★ |  | 7/7 | Good |  |
| Campbell | Cross-sectional | ★ | ★ |  | ★ | ★ | ★ | ★ |  | 6/7 | Good |  |
| Kim | Cross-sectional | ★ | ★ |  |  | ★ | ★ | ★ |  | 5/7 | Fair |  |
| Fuller-Thomson | Cross-sectional | ★ |  |  | ★ | ★ | ★ | ★ |  | 5/7 | Fair |  |
| Wang (2009) | Cross-sectional | ★ | ★ |  | ★ | ★ | ★ | ★ |  | 6/7 | Good |  |
| Ratcliffe | Cross-sectional | ★ |  | ★ | ★ | ★ | ★ | ★ |  | 6/7 | Good |  |
| Wang (2007) | Cross-sectional | ★ |  | ★ |  | ★ | ★ | ★ |  | 5/7 | Fair |  |
| Breslau | Cross-sectional | ★ | ★ |  |  | ★ | ★ | ★ |  | 5/7 | Fair |  |

The table shows the results of Newcastle-Ottawa-scale (NOS) or a modified NOS (JAMA Neurol 2019;76:144–151), performed for nonrandomized included studies. A ‘good’ quality score required three or four stars in selection, one or two stars in comparability, and two or three stars in outcomes. A ‘fair’ quality score required two stars in selection, one or two stars in comparability, and two or three stars in outcomes. A ‘poor’ quality score reflected no or one star(s) in selection, or no stars in comparability, or no or one star(s) in outcomes

For cross-sectional studies: 1—representativeness of the sample; 2—sample size adequacy; 3—non-respondents; 4—ascertainment of the exposure (risk factor); 5—comparability in different outcome groups based on the study design or analysis; 6—assessment of the outcome; 7—statistical test

For case–control studies: 1—case definition adequacy; 2—representativeness of the cases; 3—selection of controls; 4—definition of controls; 5—comparability of cases and controls on the basis of the design or analysis; 6—ascertainment of exposure; 7—same method of ascertainment for cases and controls; 8—non-response rate

For cohort studies: 1—representativeness of the exposed cohort; 2—selection of the non-exposed cohort; 3—ascertainment of exposure; 4—demonstration that outcome of interest was not present at start of study; 5—comparability of cohorts on the basis of the design or analysis; 6—assessment of outcome; 7—follow-up long enough for outcomes to occur; 8—adequacy of follow-up of cohorts
